# Evaluating the effect of xylitol wipes on cariogenic bacteria in infants and toddlers: a longitudinal clinical trial

**DOI:** 10.1186/s12903-026-08018-x

**Published:** 2026-03-27

**Authors:** Laila M. El–Habashy, Omar Abd El Sadek El Meligy, Aliaa Abdelsalam Hamouda

**Affiliations:** 1 Pediatric Dentistry and Dental Public Health Department, Faculty of Dentistry, Alexandria University, Alexandria, 21131 Egypt; 2 Pediatric and Community Dentistry Department, Faculty of Dentistry, Pharos University, Alexandria, Egypt

**Keywords:** Clinical trial, Early childhood caries, Lactic acid, Mutans streptococci, Xylitol wipes

## Abstract

**Background:**

Xylitol is widely used in caries prevention and has been shown to reduce cariogenic activity in children. However, limited evidence exists regarding the effectiveness of xylitol wipes in infants and toddlers.

**Objective:**

To investigate the effect of xylitol wipes on cariogenic activity in infants and toddlers.

**Materials and methods:**

This prospective, longitudinal, single–arm interventional study included 20 healthy, caries–free children aged 9 months to 1.5 years. Mothers were instructed to wipe their children’s teeth with xylitol wipes (Spiffies Baby Tooth Wipes™) three times daily for four weeks. Salivary samples were collected at baseline and weekly follow–up visits. Cariogenic activity was assessed by measuring lactic acid production using a chairside kit (Clinpro Cario L–Pop), and salivary mutans streptococci (MS) levels were determined using the CRT bacteria caries risk test.

**Results:**

A highly significant reduction in salivary lactic acid levels was observed after one week of xylitol wipe use (*P* < 0.0001). A significant reduction in MS counts was observed by the fourth week (*P* = 0.02).

**Conclusions:**

Daily application of xylitol wipes reduced cariogenic activity in young children, suggesting that this delivery method may be a practical preventive approach during early childhood.

**Trial registration:**

This trial was registered at ClinicalTrials.gov (Identifier: NCT07245433) on 14 November 2025.

## Background

Dental caries is a biofilm–mediated, diet–modulated, multifactorial disease characterized by the progressive demineralization of dental hard tissues due to organic acids produced by bacterial fermentation of dietary carbohydrates. Contemporary models of caries pathogenesis emphasize the ecological plaque hypothesis, in which frequent sugar exposure leads to a shift in the composition and metabolic activity of the oral biofilm toward acidogenic and aciduric microorganisms [1]. Children with early mutans streptococci (MS) colonization have a higher risk of developing caries than those with later colonization or none [2]. Prevention or delay of MS and LB colonization may be advantageous for the prevention of early childhood caries (ECC) [1, 2]. Therefore, preventive strategies aimed at reducing cariogenic bacterial activity at an early stage are of particular importance in infants and toddlers, especially those at risk of ECC.

Multiple investigations in both pediatric and adult populations have reported notable decreases in dental caries following regular intake of xylitol [3–9]. Xylitol interferes with the metabolic pathways of MS, triggering an energetically wasteful cycle that ultimately results in cell death [4], and has been associated with reduced bacterial adherence, decreased acidogenicity, and diminished virulence [5–9]. Several studies have demonstrated sustained decreases in MS counts in saliva and plaque following regular exposure to xylitol [10–15], although some studies have reported limited long–term reductions [16, 17]. Evidence also suggests that xylitol may be particularly effective during periods of tooth eruption [18, 19], and maternal consumption has been associated with reduced transmission of cariogenic bacteria and lower caries risk in children [20, 21].

Preventive regimens using xylitol should be individualized and periodically reassessed to monitor changes in caries risk status [22, 23]. Current evidence indicates that a total daily intake of approximately 3–8 g of xylitol is required to achieve a measurable clinical effect when delivered in commonly used forms [24–26]. However, delivery methods remain an important consideration in infants and toddlers, for whom commonly used forms such as chewing gum or lozenges may be unsuitable due to safety or compliance considerations [21]. Alternative delivery approaches, including syrups, lozenges, and slow–release devices, have demonstrated reductions in caries incidence or bacterial levels in children [26–29].

A more recent modality is xylitol wipes, which may provide both mechanical cleaning and topical exposure to xylitol. Zhan et al. [30] reported that the use of xylitol wipes resulted in a significant reduction in new decayed surfaces after one year, although no significant change in MS counts was observed.

Despite extensive evidence supporting the anti–cariogenic effect of xylitol, limited data are available regarding delivery methods suitable for infants and toddlers, particularly with respect to xylitol wipes and their short–term effects on cariogenic activity. Therefore, the present study was designed to evaluate the effect of xylitol wipes on cariogenic bacteria in infants and toddlers by measuring their effect on salivary MS count and lactic acid levels.

The null hypothesis of this study was that there would be no difference in salivary MS count or lactic acid level before and after xylitol wipe application in infants and toddlers.

## Materials and methods

### Ethical approval

Ethical approval for this study was obtained from the Faculty of Dentistry, Alexandria University Ethical Committee (IRB No. 00010556–IORG 0008839). The Manuscript Ethics Committee approval number was 1185–10/2025.

### Study design and setting

This study was conducted as a prospective, longitudinal, single–arm interventional trial. The TREND (Transparent Reporting of Evaluations with Nonrandomized Designs) statement guidelines were followed throughout the study.

A total of 20 healthy children aged 9–18 months, attending the Pediatric Dentistry and Dental Public Health Department, Faculty of Dentistry, Alexandria University, were enrolled.

### Sample size calculation

Sample size was estimated assuming a 5% alpha error, 80% study power, and a 95% confidence level. Kayalvizhi et al. [31] reported mean (SD) Streptococcus mutans (S. mutans) counts of 3.24 (4.90) and 1.71 (3.54) before and after xylitol wipe use, respectively, with a mean difference of 1.53 (6.11) and a 95% CI of − 5.73 to 2.97. Based on the comparison of paired means, the minimum required sample size was calculated to be 18, which was increased to 20 to compensate for potential loss to follow–up. Calculations were performed using MedCalc Statistical Software version 19.0.5 (MedCalc Software bvba, Ostend, Belgium; https://www.medcalc.org; 2019).

### Eligibility criteria

The sample was obtained by a single experienced pediatric dentist who examined all children. Only those meeting the inclusion criteria were enrolled.

### Inclusion criteria

The study included healthy children with no systemic diseases or special health care needs, aged 9–18 months. Participants were selected from moderate socioeconomic backgrounds, determined by the mother’s level of education. Only caries–free children were eligible, and all enrolled children had mothers who served as their primary caregivers for more than eight hours per day. Mothers were required to provide informed consent and demonstrate willingness to comply with study instructions.

### Exclusion criteria

Children or mothers with a history of antibiotic use within the previous month were excluded due to the potential alteration of oral flora. Children taking antihistamines were also excluded, as these medications may affect salivary flow. Additional exclusion criteria included a history of gastrointestinal conditions such as irritable bowel syndrome, as well as any known allergy or hypersensitivity to xylitol.

### Interventions

At baseline, written informed consent was obtained from the mothers prior to enrollment, after the study objectives, procedures, and their right to withdraw at any time without consequence had been explained. A thorough dental examination was performed for each infant, and a dental chart was prepared to document the erupted teeth. Dietary habits were recorded to help correlate any unexpected outcomes during the study.

A total of 84 boxes of xylitol wipes (Spiffies Baby Tooth Wipes™, Dr. Products Inc., Tucson, AZ, USA), each containing 20 disposable wipes, were used in the present study. A tooth wipe is a durable towelette infused with a xylitol–containing solution. It offers dual benefits by physically removing plaque and delivering xylitol into the oral cavity as it is used. Three flavors were available: grape, mango, and apple.

Instructions for using the wipes were explained to the mothers according to the manufacturer’s instructions. The wipe was secured around the index finger and held in place with the thumb, then applied to the teeth and gingiva using a gentle scrubbing motion. Mothers were instructed to wipe their children’s teeth three times daily after meals for 4 weeks (28 days), which provided a therapeutic dose of xylitol equivalent to approximately 4.2 g/day [30].

To monitor compliance, a timetable chart was provided to each mother. The chart represented the 7 days of the week, and each day included three boxes corresponding to breakfast, lunch, and dinner. Mothers were instructed to tick the appropriate box after each application. In addition, parents were contacted regularly by telephone to reinforce adherence to the study protocol and to address any concerns. The study duration was relatively short, and parents were informed of the importance of adherence; all participating families were cooperative, and all participants completed the study with no loss to follow–up.

### Study outcome assessment

#### Baseline sample

**Fig. 1:**
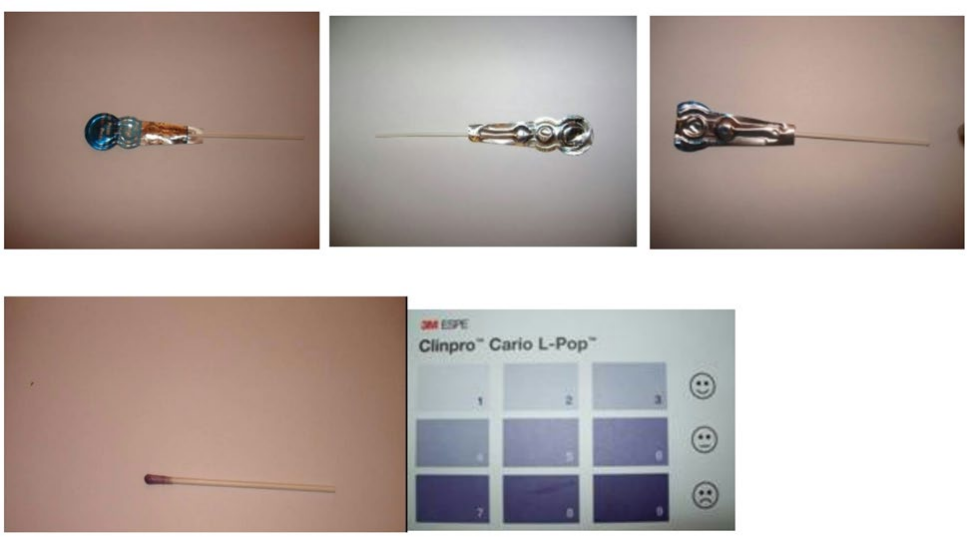
Measurement of salivary lactic acid using the Clinpro Cario–L–Pop test, showing activation of the device and interpretation of the colorimetric scale

**Fig. 2:**
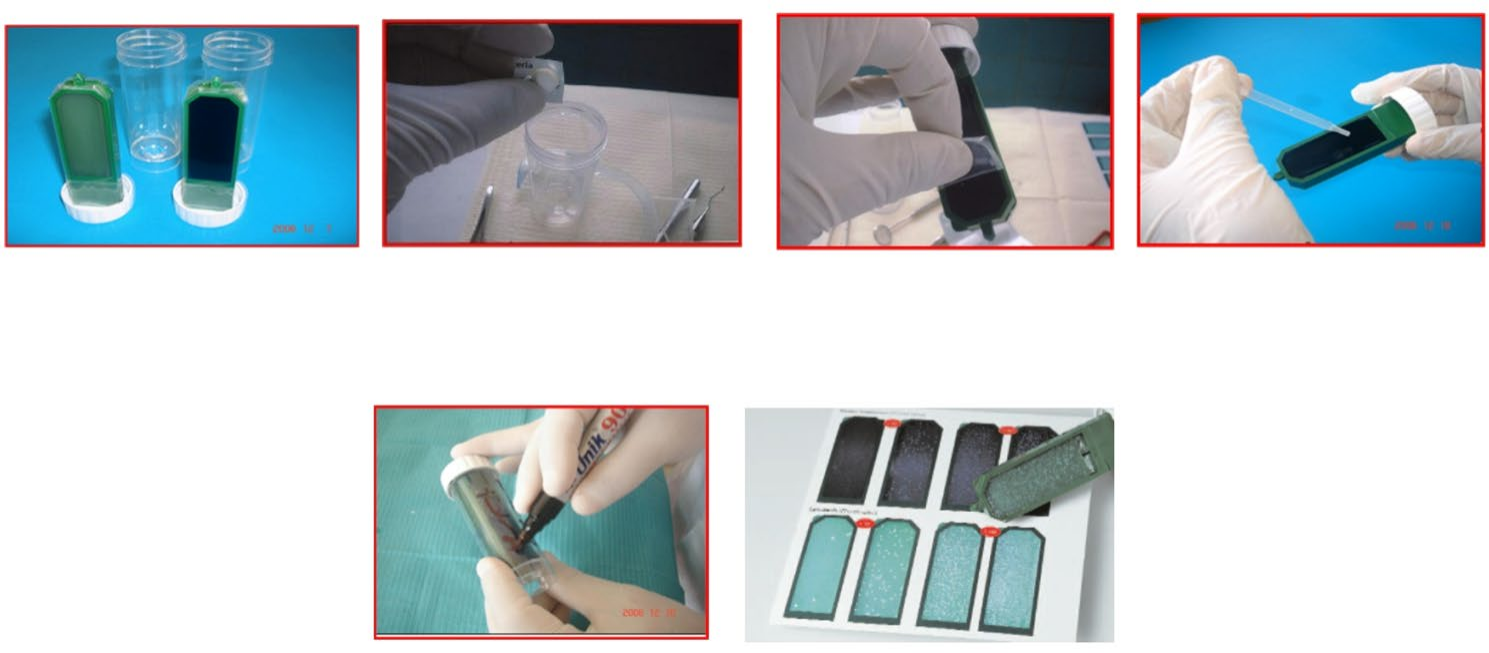
Assessment of mutans streptococci (MS) using the CRT bacteria caries risk test, including inoculation, incubation, and evaluation of bacterial growth

1. Measuring salivary lactic acid level: Salivary lactic acid levels were measured using the Clinpro Cario–L–Pop test (3 M ESPE AG Dental Product, Germany), a chair–side biochemical method used to assess lactic acid levels in oral samples according to the manufacturer’s instructions. The indicator swab provided with the kit was used to obtain the oral sample and was subsequently inserted into the blister containing the diagnostic reagents. The method is based on the enzymatic oxidation of lactic acid to pyruvate by lactate dehydrogenase, coupled with a cascade of redox indicators that produce a purple–blue color within 2 min. The resulting color was compared with a standardized nine–field color chart, where scores of 1–3 indicate low lactic acid production, 3–6 indicate moderate production, and 7–9 indicate high lactic acid production (Fig. 1).
2. Measuring MS count: For detection of MS the chair side test “CRT bacteria caries risk test” (ivoclar vivadent AG, fl–9494 schaan/liechtenstein) was used. This test enables the simultaneous determination of the MS and LB counts in saliva by means of selective agars. The blue mitis–salivarius–agar with bacitracin is used to detect MS, while the light culture medium, Rogosa agar, is used to evaluate LB [32, 33]. In the present study, only the blue mitis–salivarius–agar with bacitracin was used to detect MS. The evaluation model chart represents none, mild, moderate and severe scores respectively (Fig. 2).

Saliva samples were collected from each child using the passive drooling method, in which a small sterile chin cup was used to guide naturally pooled saliva into a collection tube. The agar carrier was removed from the test vial, and NaHCO_3_ was placed at the bottom of the test vial which releases CO_2_ when it comes in contact with moisture, creating a favorable condition for bacterial growth. The protective foils were carefully removed from the agar surfaces without touching the agar, and the agar surface was thoroughly wetted with saliva using a pipette, allowing excess saliva to drip off. The agar carrier was then returned to the vial, which was tightly closed and incubated at 37 °C in the CRT incubator for 48 h. The density of MS colonies was compared with the corresponding evaluation pictures in the enclosed model chart, and scores of 0, 1, 2, and 3 were assigned to none, mild, moderate, and severe growth, respectively (Fig. 2).

### Follow–up sessions

Mothers and infants were recalled every week for 4 weeks.

To assess compliance in using the wipes properly and on scheduled time, to check any changes in dietary habits and to check any side effects form xylitol that include diarrhea, allergy, gas, or others. Follow–up samples were taken in each visit as previously described where, each child was tested for salivary lactic acid level using the Clinpro Cario–L–Pop, and for the streptococci count using CRT.

### Data analysis

Descriptive statistics were calculated for qualitative variables as frequencies and percentages whereas mean and standard deviation were calculated for quantitative variables. Differences between MS scores in various visits were assessed using Wilcoxon signed ranks test since the variable was ordinal. Differences between scores were recoded to indicate (1) increase in score, (2) score remaining unchanged and (3) decrease in score. The three categories were compared across the various follow–up periods using Freidman test. Normality of lactic acid scores was checked and established using Kolmogrov Smirnov test. Repeated measures analysis of variance was used to assess the differences between these scores across time with Greenhouse– Geisser correction for violation of sphericity followed by Bonforonni adjustment for comparison of pairs. Significance was set at the 5% level. SPSS version 17.0 was used for statistical analysis. Line graphs were used to plot change in scores across time whereas bar charts were used to display the distribution of change in MS scores across time. A bar of pie was used to represent the undesired effects experienced by the children when they used the wipes.

## Results

**Fig. 3:**
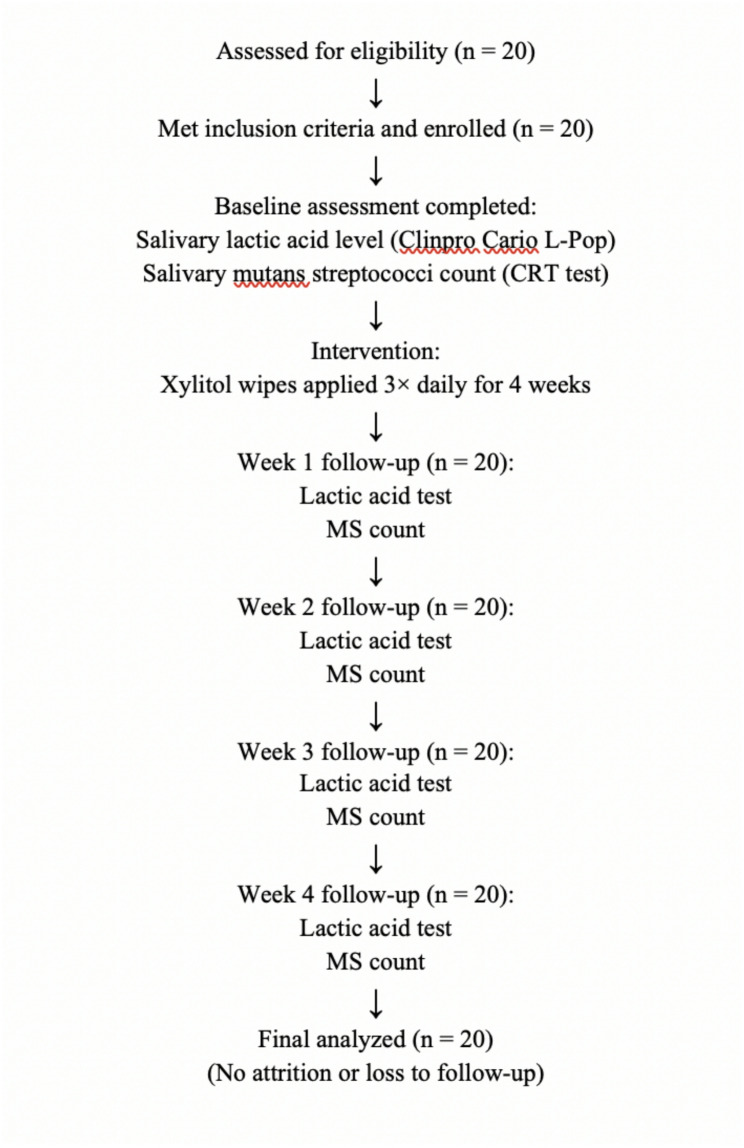
Flow diagram of participant enrollment, intervention, follow–up, and analysis during the study period, in accordance with TREND guidelines

A flow chart illustrating participant enrollment and follow–up is presented in Fig. 3, in accordance with the TREND guidelines, which recommend documenting the flow and attrition of participants throughout longitudinal studies.

In the present study, the effect of xylitol wipes on cariogenic bacteria in 20 young children was evaluated with respect to: (a) lactic acid production measured using the Clinpro Cario L–Pop kit; (b) MS counts assessed with the CRT kit; and (c) parent/child acceptance of the wipes.

Data was collected at baseline session and then on four follow–up consecutive visits with one–week interval.

This study included 20 infants of mean (SD) age = 13.4 (2.9), ranging between 9 and 18 months. Children who were one–year old or younger represented 55% of the study sample.

**Fig. 4:**
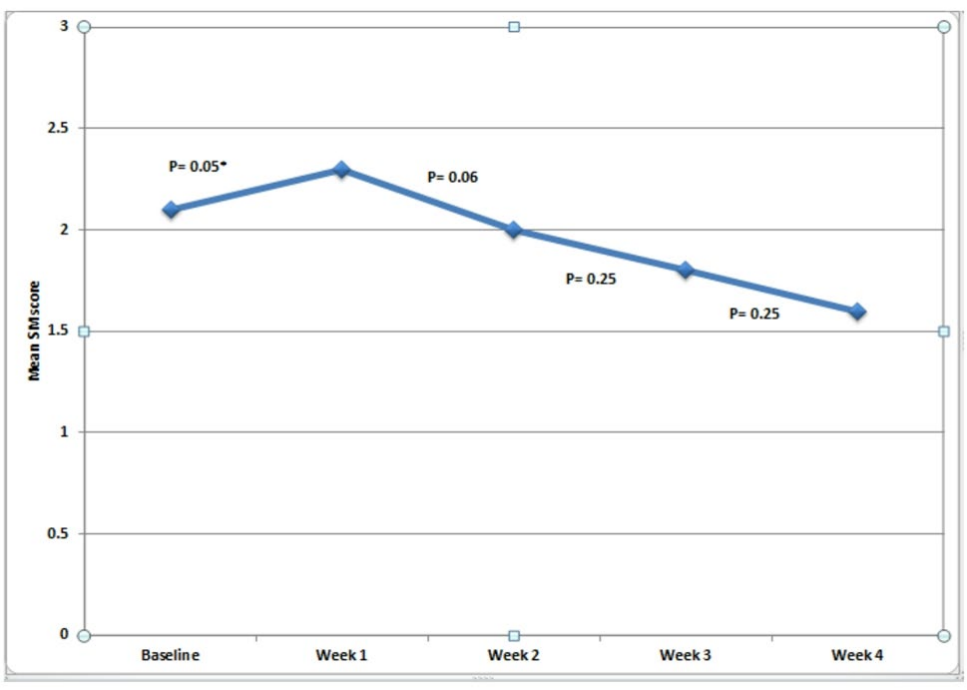
Changes in mean mutans streptococci (MS) scores from baseline to week 4 (*: Statistically significant at *P* ≤ 0.05)

**Fig. 5:**
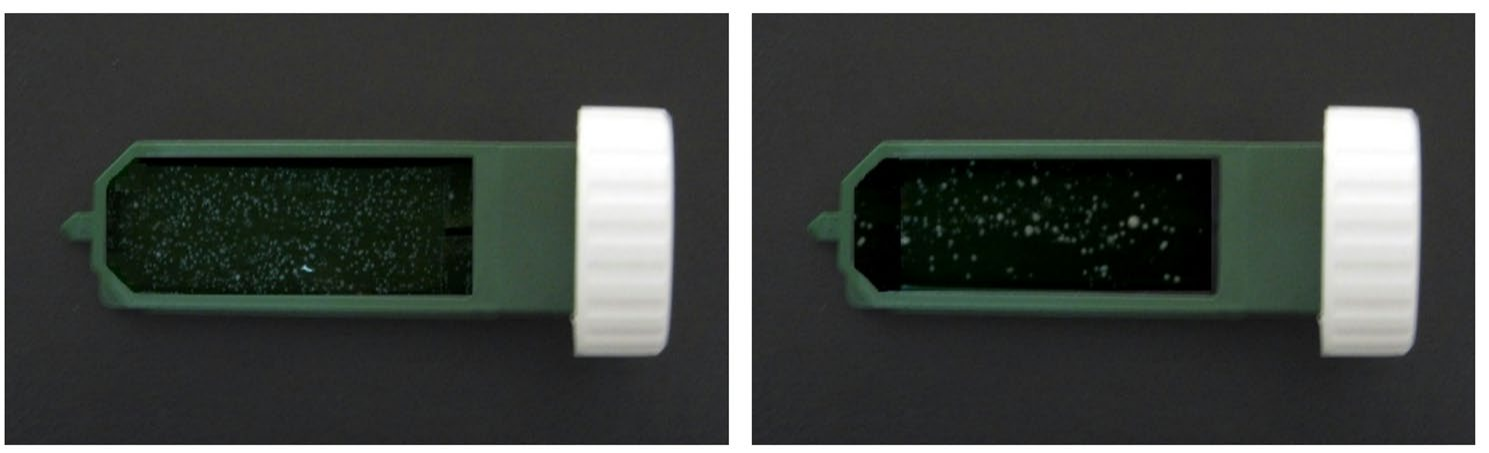
Representative CRT cultures showing mutans streptococci (MS) growth at baseline (medium growth, left) and at week 4 (mild growth, right)

The mean (SD) MS score increased significantly one week after baseline (2.1 ± 0.7 and 2.3 ± 0.7, *P* = 0.05). The score then began to decrease, though not significantly, at weeks 2 and 3 (2.0 ± 0.5 and 1.8 ± 0.8, respectively; *P* = 0.06 and 0.25), followed by a further decline at week 4 (1.6 ± 0.5, *P* = 0.25). Compared to baseline, the week 4 score was significantly lower (*P* = 0.02), whereas the scores after weeks 2 and 3 were not significantly lower (*P* = 0.41 and 0.06, respectively) (Figs. 4 and 5).

**Fig. 6:**
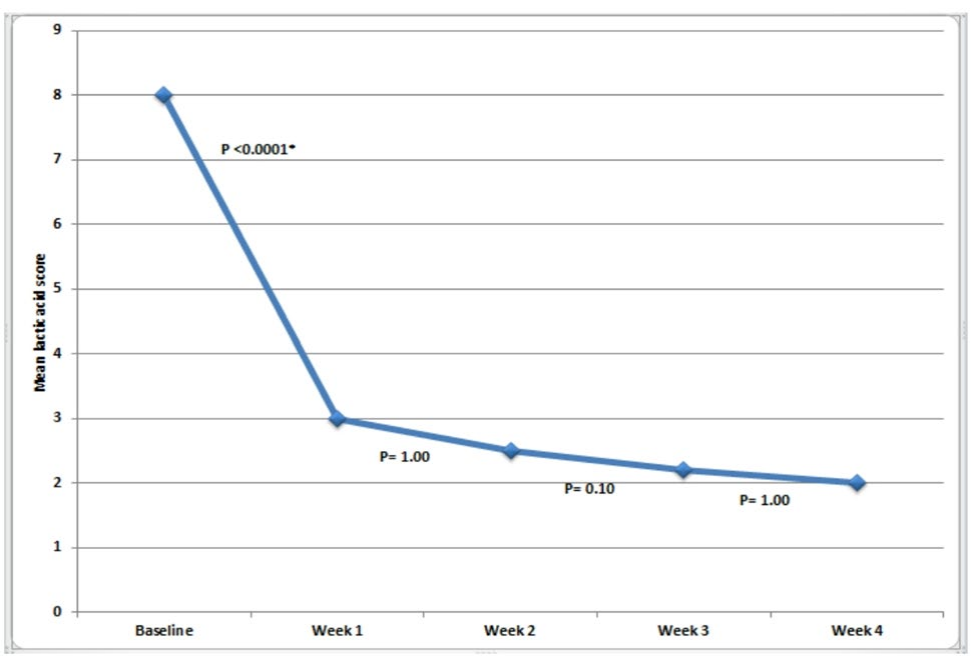
Changes in mean salivary lactic acid scores from baseline to week 4 (*: Statistically significant at *P* ≤ 0.05)

**Fig. 7:**
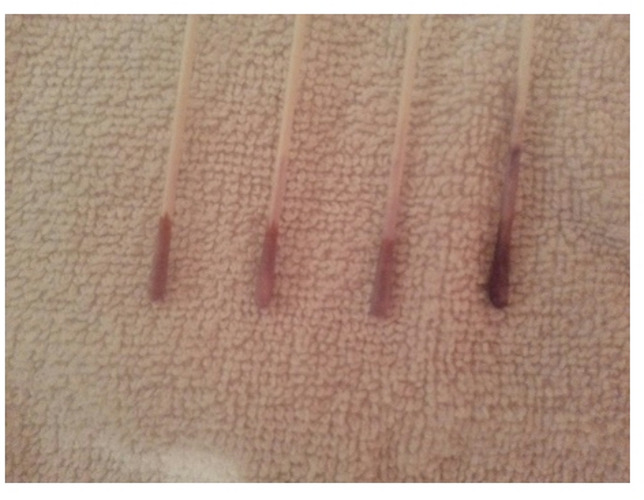
Representative color changes of the lactic acid test corresponding to different levels of lactic acid production observed during the study period

The mean ± SD lactic acid score decreased from 8.0 ± 0.8 at baseline to 3.0 ± 1.2, 2.5 ± 0.7, 2.2 ± 0.9, and 2.0 ± 0.9 at weeks 1, 2, 3, and 4, respectively. The reduction from baseline to week 1 was statistically significant (*P* < 0.0001), whereas reductions across the subsequent follow–up periods were not statistically significant (*P* = 1.00, 0.10, and 1.00 for weeks 1–2, 2–3, and 3–4, respectively). Baseline scores were significantly higher than scores at all follow–up visits (*P* < 0.0001 for all). No significant differences were observed among scores at weeks 1, 2, and 3, while the week 4 score was significantly lower than the week 1 score (*P* = 0.008) but not significantly different from weeks 2 and 3 (*P* = 0.66 and 1.00, respectively) (Figs. 6 and 7).

All parents reported acceptance of the wipes and even found it easier than brushing their infants’ teeth since it was easier to control and manage reaching all teeth surfaces. None of them reported missing a wipe.

Most children (15, 75%) accepted the wipes. All 5 children who indicated non–acceptance in the first week later reported improved acceptance. Out of the five children who did not accept the wipes in the first week, two were reported by their parents to have flatulence and mild diarrhea which disappeared after the first week and one was reported to have flatulence only that similarly disappeared after the first week.

## Discussion

Twenty children were enrolled in the present study who ranged in age from 9 to 18 months. This age group was selected as it is the most critical age for developing early childhood caries [30]. It is also the most difficult age for parents to control the child to perform good brushing.

Accordingly, xylitol wipes would provide a good prevention technique from caries to the newly erupted teeth with immature enamel calcification. Evidence also showed that xyliol works most effectively on newly erupted teeth [26, 30, 34].

Each child was given a total of 84 wipes to be used 3 per day after each main meal till the end of the study, by this way each child received a total dose of 4.2 g daily achieving the safe therapeutic dose of caries prevention according to the AAPD [21].

Because acidogenic oral microorganisms produce acids when fermenting available carbohydrates, their metabolic activity plays a key role in dental caries development; increased activity corresponds to a greater cariogenic potential. Therefore, Clinpro Cario L–Pop was used in the present study to provides an easy and accurate chairside procedure to measure the lactic acid of the cariogenic bacteria. Research indicates that lactate–dehydrogenase–based enzymatic assays more effectively detect a broad spectrum of acid–producing microorganisms implicated in caries, including both MS and non–MS species. This approach demonstrates greater accuracy than diagnostic methods relying on semi–synthetic media with sucrose, tryptose, and yellow–purple indicators [35–40].

The chair side test CRT bacteria caries risk test was used in the present study to measure MS count as it provides a significant simplification of cultural procedures by eliminating the need for media preparation and saliva processing procedures allowing for an easy and time–saving method which is particularly useful when diagnosing small children [41, 42]. Moreover, the comparison of CRT bacteria with laboratory methods showed that CRT bacteria proved to reacts more selectively compared with conventional MSB agar, it also reacted more sensitively and was able to detect even low bacterial counts, which permitted early detection of MS [32, 33].

The present study demonstrated a significant reduction in MS counts by the fourth week of using xylitol wipes in children. These findings are consistent with previous research employing different xylitol delivery forms, which also reported significant reductions in streptococcal levels within approximately three weeks of use [6, 12, 21]. On the other hand, other studies have not shown long term reduction in MS with xylitol use in different forms, but all showed reduction in caries rates [15, 16, 30]. The mechanism by which xylitol reduces caries in these studies was not fully understood, and different explanations have been suggested: there was a trend toward transient changes in the genotype of MS in the xylitol group; some researchers proposed that xylitol affected other cariogenic bacteria besides MS; and others suggested that xylitol altered MS by reducing their virulence. The anti–cariogenic benefits of xylitol were further demonstrated by Zhan, et al. [30], who reported a marked reduction in new carious lesions among young children (6–35 months) treated with xylitol wipes over a one–year period compared with a placebo group. Although caries incidence decreased, the authors did not observe a corresponding decline in S. mutans counts. MS levels remained low during the first six months and then rose sharply by the one–year mark. The investigators attributed this pattern to the natural course of oral MS colonization, which can begin in predentate infants, increases following eruption of the primary dentition, and typically peaks between 13 and 21 months before continuing to rise. In their cohort, children were 6–35 months old at baseline and 18–47 months old after one year. These developmental factors likely contributed to the pronounced increase in MS levels at the one–year follow–up and highlight the vulnerability of children in this age range to early caries development.

The result of the present study showed that during the first, second, and 3rd week of using the wipes no significant reduction occurred in the MS count but in the 4th week there was significant reduction noted. The explanation for gradual reduction of MS count can be attributed to the fact that xylitol doesn’t kill bacteria immediately. Xylitol tricks plaque bacteria so that when the organism try to metabolize it, they convert it to xylitol 5 phosphate, which is toxic to them. In expelling this substance, they waste energy they would otherwise use to grow and multiply. In other words, the bacteria try to metabolize xylitol but can’t get fuel from it, replacing sucrose with xylitol starves the bacteria which gradually dies, that is why the significant reduction in the streptococci count was encountered in the present study only by the 4th week [4, 9–11].

The findings of this study revealed a highly significant reduction in lactic acid production by cariogenic bacteria, with scores decreasing from severe (score 8) to mild (score 3) beginning in the first week of xylitol wipe use. These values continued to drop through the rest of the four follow–up visits from score 3 at the first follow–up visit to 2.5, 2.2, 2 respectively. These values represent mild lactic acid production according to the Clinpro Cario–L–Pop scale as in this region the lactic acid is very week to decalcify the tooth structure [3, 4]. That is to say, the peak action of the xylitol wipes in reducing lactic acid production occurred during the first week of use and was maintained low by the continuation of using the wipes.

The interpretation of the rapid drop in lactic acid production in the present study is in accordance with other studies which emphasized that cariogenic bacteria do not metabolize xylitol and thus, no lactic acid is produced [10, 11]. Since lactic acid is the most important indicator for cariogenic activity [38, 39, 43], the findings of the present study support the anti–cariogenic property of the xylitol wipes [30].

In the current study the xylitol wipes showed to be very convenient to both parents and children as they have a pleasant, sweet taste that appealed to the children as reported by parents. Moreover, the tooth wiping experience was found to be easily controlled by parents to reach all tooth surfaces these findings were in accordance with Zahan et al. [30] However, mild gastrointestinal tract (GIT) symptoms in the form of transient diarrhea and increased flatulence were reported in three cases during the first week, which resolved spontaneously. A similar finding has been reported in a previous study [21].

### Limitations

This study is limited by the absence of randomization and the lack of a control group. In addition, the short follow–up period may have restricted the ability to observe changes that could emerge over a longer duration, particularly as more teeth erupt. Dietary habits were recorded but were not analyzed in relation to the study outcomes; this factor may have influenced cariogenic activity and should be considered in future studies.

The null hypothesis of this study was rejected, as significant differences were observed in salivary lactic acid levels and mutans streptococci counts after the use of xylitol wipes in infants and toddlers.

## Conclusions

Based on the results of this study, it can be concluded that:

1. Xylitol wipes exert an anti–cariogenic effect by lowering MS levels and reducing lactic acid production by cariogenic bacteria.
2. Xylitol wipes are well–accepted by children due to their pleasant taste.
3. They also provide parents with a simple, convenient, and effective method for cleaning their children’s teeth.

## Recommendations

It appears from the result of the present study that xylitol wipes significantly reduced MS count and lactic acid production of cariogenic bacteria in infant and toddlers, xylitol wipes can be an effective method in reducing ECC especially in moderate to high–risk patients. Xylitol wipes should be advised as adjunct tools besides tooth brushing in preventing caries in children.

## Data Availability

The datasets used and/or analyzed during the current study are available from the corresponding author on reasonable request.

## Abbreviations

FDA: Food and Drug Administration
AAPD: American Academy of Pediatric Dentistry
MS: Mutans streptococci
LB: Lactobacilli
ECC: Early childhood caries
AAP: American Academy of Pediatrics
TREND: Transparent Reporting of Evaluations with Nonrandomized Designs
S. mutans: Streptococcus mutans
CRT: Caries Risk Test
GIT: Gastrointestinal tract
